# Application of Redox-Responsive Hydrogels Based on 2,2,6,6-Tetramethyl-1-Piperidinyloxy Methacrylate and Oligo(Ethyleneglycol) Methacrylate in Controlled Release and Catalysis

**DOI:** 10.3390/polym13081307

**Published:** 2021-04-16

**Authors:** Miriam Khodeir, He Jia, Alexandru Vlad, Jean-François Gohy

**Affiliations:** Institute of Condensed Matter and Nanosciences (IMCN), Université catholique de Louvain, Place L. Pasteur 1, B-1348 Louvain-la-Neuve, Belgium; miriam.khodeir92@gmail.com (M.K.); he.jia@uclouvain.be (H.J.); alexandru.vlad@uclouvain.be (A.V.)

**Keywords:** hydrogels, redox-responsive polymers, TEMPO, encapsulation-release, catalysis

## Abstract

Hydrogels have reached momentum due to their potential application in a variety of fields including their ability to deliver active molecules upon application of a specific chemical or physical stimulus and to act as easily recyclable catalysts in a green chemistry approach. In this paper, we demonstrate that the same redox-responsive hydrogels based on polymer networks containing 2,2,6,6-tetramethyl-1-piperidinyloxy (TEMPO) stable nitroxide radicals and oligoethylene glycol methyl ether methacrylate (OEGMA) can be successfully used either for the electrochemically triggered release of aspirin or as catalysts for the oxidation of primary alcohols into aldehydes. For the first application, we take the opportunity of the positive charges present on the oxoammonium groups of oxidized TEMPO to encapsulate negatively charged aspirin molecules. The further electrochemical reduction of oxoammonium groups into nitroxide radicals triggers the release of aspirin molecules. For the second application, our hydrogels are swelled with benzylic alcohol and tert-butyl nitrite as co-catalyst and the temperature is raised to 50 °C to start the oxidation reaction. Interestingly enough, benzaldehyde is not miscible with our hydrogels and phase-separate on top of them allowing the easy recovery of the reaction product and the recyclability of the hydrogel catalyst.

## 1. Introduction

Hydrogels are three-dimensional, hydrophilic, polymeric networks capable of swelling in aqueous medium and resembling to some extent living tissues [1]. They may be chemically stable or they may degrade or dissolve under specific conditions [2]. When the polymer networks are held together via polymer chain entanglements, crystallites or non-covalent interactions including hydrophobic interactions, Van der Waals interactions, hydrogen bonds and ionic forces, they are referred to as physical gels [3]. The so-called permanent or chemical hydrogels are obtained from covalently-crosslinked networks and generally present a better homogeneity than physical gels [2]. Since the seminal publication of Wichterle and Lim [4], chemical hydrogels have gained attention owing to their unique characteristics, especially when they are originating from stimuli-responsive networks that can dynamically and reversibly alter their structure and properties in response to changes in the environment [5]. Hydrogels are utilized in many areas and more specifically in the biomedical field where they can act as drug protectors, targetable carriers for bioactive drugs etc. [2,3,6,7]. As far as biomedical applications are concerned, hydrogels derived from bio-based polymers are particularly attracting since those polymers are generally biocompatible and biodegradable and often show a high level of biomimicry, a highly desired characteristic for in vivo applications [8]. Moreover, in order to reach the desired properties with ever increasing complexity, polymer-based hydrogels may be mixed with other polymeric or non-polymeric components to form composite hydrogel systems, including e.g., polymer nanoparticles, electrospun fibers, nanocarbons, etc. [8,9,10].

In the present contribution, we focus on stimuli-responsive chemical hydrogels. Typical stimuli include variations in temperature [11], pH [11], applied stress [12], magnetic and electromagnetic field [13], ionic strength [14], light [15] and the presence of bioactive species [16]. Redox-responsive gels have been scarcely reported. Typically, species which may undergo reversible oxidation-reduction reactions are good candidates to achieve redox responsiveness in hydrogels [17]. The design of redox active networks usually involves the incorporation of redox responsive groups either in the polymer main chain, in the side groups or as cross-linking moieties [17,18]. From a practical point of view, redox stimuli may be applied chemically or electrochemically. This last possibility is particularly promising since the addition of reactants to the hydrogel is not required in order to observe the redox-responsive behavior. However, the hydrogel should display a sufficient electric conductivity in order to be electrochemically addressed.

As far as redox groups are concerned, 2,2,6,6-tetramethyl-1-piperidinyloxy (TEMPO) is a very interesting candidate. TEMPO is a stable nitroxide radical that can be easily oxidized into an oxoammonium cation or reduced into an aminoxyl group [19]. Moreover, TEMPO derivatives are largely used in chemistry as catalysts [20], and in the biomedical field as imaging enhancers in electron spin resonance techniques or as a radical scavenger of reactive oxygen species and are, therefore, considered valuable candidates for anti-oxidant therapies [21]. Moreover, the redox equilibrium associated with nitroxide radicals, and especially TEMPO, has been recently used for the production of energy storage devices [22,23]. For the energy storage application, scientists developed a poly(methacrylate) bearing TEMPO moieties as side groups. In our recent previous works [24,25], we have described and characterized new redox-responsive hydrogels based on polymer networks containing the TEMPO-containing methacrylate and oligoethylene glycol methyl ether methacrylate (OEGMA). TEMPO groups can undergo reversible oxidation-reduction reactions that lead to redox activity while OEGMA groups are hydrophilic and allow water swelling in order to obtain hydrogels. In the present contribution, the use of those hydrogels for two different applications is presented.

The first application deals with the release of a guest molecule, namely aspirin, in response to an electrochemical redox trigger. The advantage of the electrochemical stimulus is that it can be localized in time, it does not require the addition of reagents and the trigger parameters, such as current intensity and reaction time, and it can easily by modulated to adequately comply with the system [26,27]. Therefore, the amount of released guest molecules can be controlled and realized on demand.

The second application is based on the well-known use of TEMPO groups as catalysts for the oxidation of alcohols to obtain ketones, aldehydes or carboxylic acids [28,29]. The oxidation of alcohols using TEMPO-based catalysts is often efficient, fast, selective, realized in mild conditions and can tolerate sensitive functional groups [28,29]. However, the difficulty of catalyst recycling as well as the need for organic solvents and transition metal co-oxidants are limiting the application of TEMPO-based catalysts [30]. Here, we report a methodology using our hydrogels containing TEMPO groups as catalysts for the oxidation of benzyl alcohol into benzaldehyde in aqueous medium. The applied methodology is inspired by the previous work of Karimi et al. [31]. Such an application follows some of the principles of green chemistry since water is used as solvent and no metallic co-catalysts are needed. Moreover, it allows a very easy purification of the product of the reaction since the latter phase separates from the hydrogel. Therefore, the TEMPO-containing hydrogel can be easily recycled after the reaction to be used again.

## 2. Materials and Methods

### 2.1. Materials

All chemicals were purchased from Sigma-Aldrich (Overijse, Belgium), Acros (Geel, Belgium) and TCI (Zwijndrecht, Belgium). 2,2,6,6-Tetramethyl-1-piperidyl methacrylate (TMPM, 98%, TCI), oligo(ethylene glycol) methyl ether methacrylate (OEGMA, average molar mass of 300 g/mol, Sigma-Aldrich) and di(ethylene glycol) dimethacrylate (OEGMA_2_, 98%, Sigma-Aldrich) were purified on a AlO_x_-filtration column prior use in order to remove the inhibitor. The 2,2′-azobisisobutyronitrile (AIBN, 98% purity, Sigma Aldrich) initiator was recrystallized twice from methanol (Acros, 99%) prior use. Acetylsalicylic acid (ASA, 99.9%, Sigma-Aldrich), isopropanol (IPA, Acros, 99.5%), methanol (Acros, 99.8%), diethyl ether (Acros, 99%), NaHCO_3_ (Sigma Aldrich, 99%), Na_2_WO_4_.2H_2_O (Sigma-Aldrich, 99%), H_2_O_2_ (Sigma-Aldrich, 30 wt% solution in water), ethylene diamine tetraacetic acid disodium salt dihydrate (EDTA, Sigma Aldrich, 99%), NaClO (Sigma Aldrich, 99%), HBF_4_ (Sigma Aldrich, 48 wt% solution in water), NaClO_4_ (Sigma Aldrich, 98%), acetonitrile (ACN, 99.9%, Sigma Aldrich), formic acid (98%, Sigma Aldrich), benzylic alcohol (Sigma Aldrich, 99%) and tert-butyl nitrite (Sigma Aldrich, 90%) were used as received.

### 2.2. Synthesis of Hydrogels

**Figure 1:**
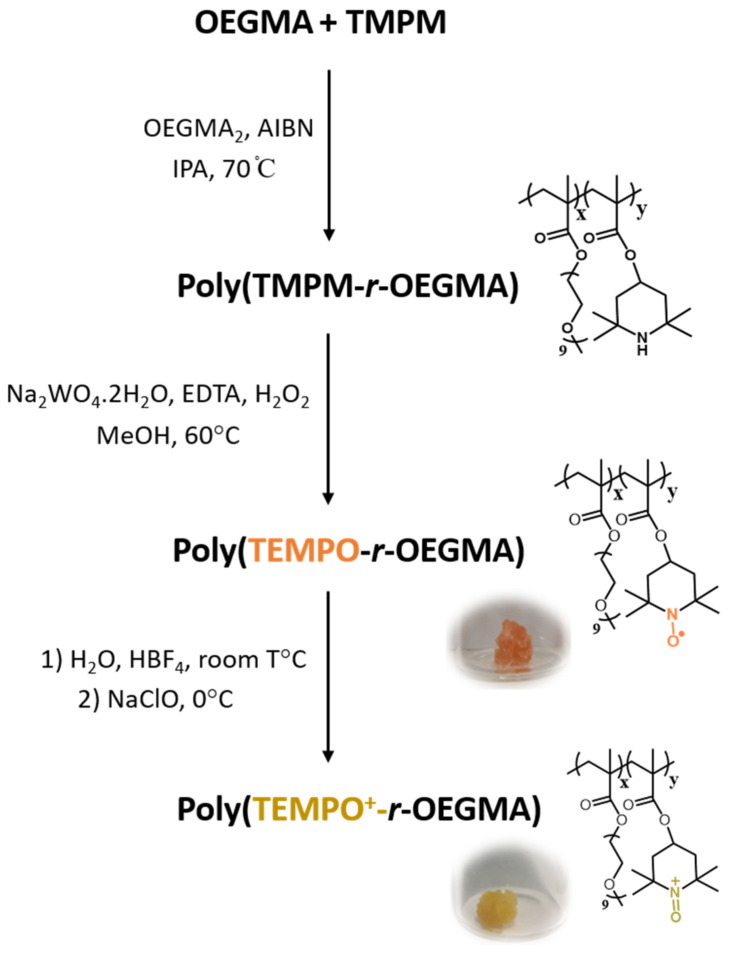
Synthesis of the poly(2,2,6,6-tetramethyl-1-piperidinyloxo ammonium methacrylate-*random*-oligo(ethylene glycol) methyl ether methacrylate) (P(TEMPO-*r*-OEGMA)) and oxidized P(TEMPO-*r*-OEGMA) (P(TEMPO^+^-*r*-OEGMA)) hydrogels.

The investigated poly(2,2,6,6-tetramethyl-1-piperidinyloxo ammonium methacrylate-random-oligo(ethylene glycol) methyl ether methacrylate), further abbreviated as P(TEMPO^+^-*r*-OEGMA), hydrogels have been synthesized via a methodology described in our previous work [24]. Briefly, TMPM was dissolved in isopropanol into a round-bottom flask. The required amount of OEGMA, OEGMA_2_ and the initiator AIBN (0.5 eq.) were then added and stirred (Figure 1). The molar ratio of TMPM/(TMPM+OEGMA) (abbreviated as X_TEMPO_) was set to 0.2 and the cross-linker molar ratio OEGMA_2_/(TMPM+OEGMA) (abbreviated as X_CL_) was set to 0.03. The solution was then degassed by three freeze pump–thaw cycles and filled with argon before stirring in an oil bath at 70 °C overnight to lead to a transparent material. The TMPM units were then oxidized into TEMPO nitroxide radicals using Na_2_WO_4_ (0.25 eq.), EDTA (0.15 eq.) and H_2_O_2_ (5 eq.) in methanol (see Figure 1). The mixture was then stirred at 60 °C overnight. An orange colored gel was obtained, washed four times with distilled water and methanol (1:1, *v*:*v*) and dried in vacuum at 40 °C overnight. An orange sticky material corresponding to dried P(TEMPO-*r*-OEGMA) was finally obtained. For the oxidation of TEMPO into TEMPO^+^ (Figure 1), the dried P(TEMPO-*r*-OEGMA) material was swollen in distilled H_2_O (31.25 eq.). HBF_4_ (1 eq.) was then slowly added at room temperature, followed by the addition of NaClO (0.5 eq.) at 0 °C and additional stirring for 1 h at 0 °C. The oxidized P(TEMPO^+^-*r*-OEGMA) obtained was washed with ice-cold 5 wt% NaHCO_3_ aqueous solution and ice-cold diethyl ether. The yellow material obtained was finally dried overnight at 40 °C in vacuum. The final hydrogels were obtained by swelling the P(TEMPO^+^-*r*-OEGMA) hydrogels in distilled water for 48 h to be sure to reach the swelling equilibrium. The excess of water was removed and the remaining hydrogel was used for further experiments.

### 2.3. Electrochemical Measurements

All electrochemical experiments were performed at room temperature using 0.1 M NaClO_4_ as supporting electrolyte on a Biologic VMP300. 5 µL of sample were dropped on the working electrode (carbon screen printed electrode DRP-110-U75, Metrohm, Antwerpen, Belgium) and, after 60 s, cyclic voltammetry was performed in the potential range that comprised between 0.2 and 0.6 V at a scan rate of 10 mV/s. Measurements were repeated 5 times to ensure reproducibility.

### 2.4. Ultra-High-Performance Liquid Chromatography (UHPLC)–Electrospray Ionization (ESI) Mass Spectrometry Analysis

An ultra-high-performance liquid chromatography (UHPLC) system (ThermoFisher Scientific, Merelbeke, Belgium) consisting of a binary pump, an automatic injector, a column oven and an Agilent (Machelen, Belgium) 1290 series ultraviolet (UV) detector was used. The separation was carried out on an Eclipse plus C18 rapid resolution high definition (RRHD) column (100 × 2.1 mm, 1.8 μm) at a flow rate of 0.2 mL/min and using an aqueous solution containing 1.0% of formic acid and 5% ACN (solvent A) and ACN containing 0.1% formic acid (solvent B). The elution program was started at 90% solvent A for 1 min, then 90% solvent B for 6 min at a flow rate of 0.2 mL/min with UV detection at 280 nm. The injection volume was kept at 5 µL for the standard and all the other samples. The detection in mass spectrometry was carried out by an electrospray ionization (ESI) jet stream Agilent (Machelen, Belgium) 6150B mass spectrometer. The analysis was carried out at 200 °C with a capillary voltage of 1500 eV and a voltage nozzle at 2000 eV. The parameters were optimized for the detection of acetylsalicylic acid as follows: nebulization pressure at 50 psi, drying gas at 4 L/min and power of fragmentary set at 75 eV. The retention time of aspirin was observed at 5.52 ± 0.1 min for MS and 5.32 ± 0.1 min for UV. An aspirin standard (Sigma Aldrich, Overijse, Belgium) was dissolved in ACN containing 0.1% formic acid. A calibration curve ranging from 15 µM (2.7 ppm) to 301 µM (54.2 ppm) was injected. The aspirin was followed in LC-MS in SIM (-) mode for m/z of 179 and 225 and in UV at 280 nm. 30 µL of samples were diluted with 100 µL of water (dilution factor 4.33). Measurements were repeated 3 times to ensure reproducibility.

### 2.5. Gas Chromatography (GC)–Mass Spectrometry (MS) Analysis

The chromatographic separation of benzaldehyde and benzylic alcohol was performed using a Thermo Scientific (Merelbeke, Belgium) TRACE 1310 GC (gas chromatograph) coupled with a Thermo Scientific (Merelbeke, Belgium) single quadrupole ISQ™ QD™ mass spectrometer (MS). The GS was equipped with a RESTEK™ Rxi^®^-5Sil MS column (*L* = 30 m, *d_c_* = 0.25 mm, and *d_f_* = 0.25 μm). The GC temperature program started at a temperature of 60 °C, ramped to 300 °C (20 °C/min, held 5 min) with a constant flow of 2 mL/min, resulting in an overall analysis time of 17 min. Injection mode with split ratio of 1/10 was used. The MS was operated with source of mass at 305 °C and full scan mode from 40 to 400 *m*/*z*. Measurements were repeated 3 times to ensure reproducibility.

### 2.6. Fourier-Transform Infrared (FTIR)

Infrared spectra were collected on a Thermo Scientific (Merelbeke, Belgium) Nicolet 6700 Fourier transform infrared (FTIR) spectrometer. For the kinetic study, a probe 6.3 mm AgX DiComp (Au, Diamond, C22) was connected to a ReactIR 15 from Mettler Toledo (Zaventem, Belgium). The probe was introduced in an Easymax reactor from Mettler Toledo (Zaventem, Belgium). Measurements were repeated 3 times to ensure reproducibility.

## 3. Results

### 3.1. Synthesis of Hydrogels

P(TEMPO^+^-*r*-OEGMA) hydrogels with a molar fraction of TEMPO^+^ monomer equal to 0.2 and a molar fraction of crosslinker equal to 0.03 were synthesized (Figure 1). Briefly, a P(TMPM-*r*-OEGMA) precursor hydrogel was first prepared by conventional radical copolymerization of TMPM, OEGMA and OEGMA_2_ (OEGMA_2_ playing the role of chemical crosslinking agent since it contains two polymerizable double bonds). This was followed by the oxidation of the secondary amine of TMPM units with H_2_O_2_ and Na_2_WO_4_ in methanol to obtain TEMPO units. Finally, the nitroxide radical units of TEMPO were oxidized into oxoammonium groups (TEMPO^+^) with NaClO in the presence of HBF_4_. This reaction can be macroscopically followed by the change of color of the hydrogel from orange to yellow (Figure 1) and is confirmed by FTIR spectroscopy with a characteristic N–O vibration at 1540 cm^−1^ for P(TEMPO-*r*-OEGMA) and a characteristic N=O vibration at 1570 cm^−1^ for P(TEMPO^+^-*r*-OEGMA).

### 3.2. Encapsulation-Release of Aspirin from P(TEMPO^+^-r-OEGMA) Hydrogels

**Figure 2:**
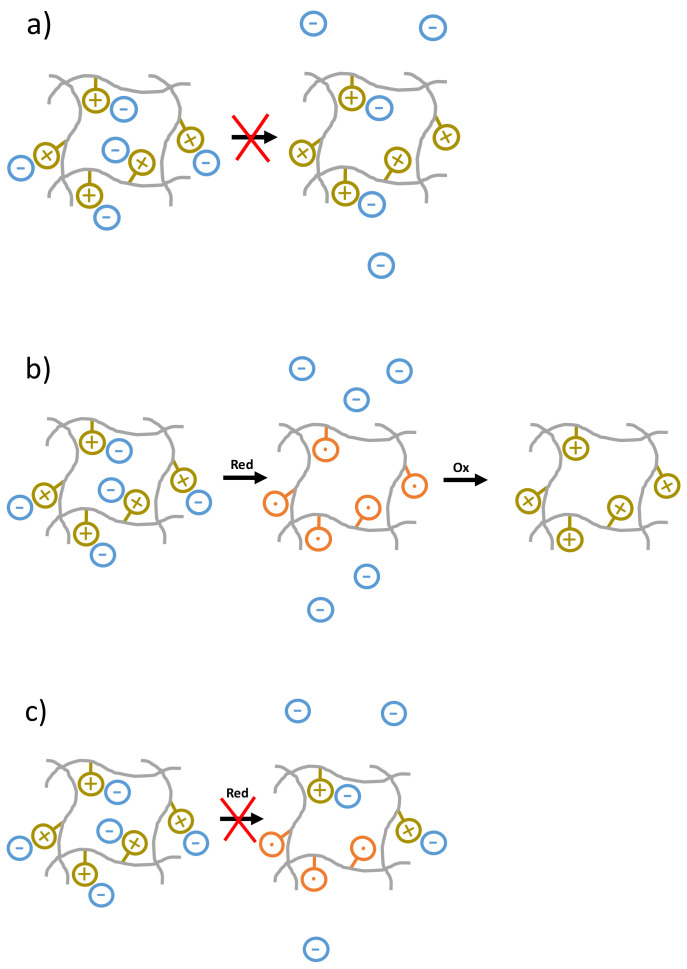
Sketches for encapsulation-release of aspirin from P(TEMPO^+^-*r*-OEGMA) hydrogels (grey lines stand for polymer chains, negatively charged aspirin molecules are represented in blue, positively charged oxoammonium are represented in yellow-greenish and nitroxide radicals are in orange). (**a**) Firmly attached aspirin do not diffuse out of the positively charged oxidized hydrogel. (**b**) The whole aspirin molecules are released from the hydrogel upon electrochemical reduction and the re-oxidized hydrogel contains no longer aspirin molecules. (**c**) Incomplete release (e.g., due to incomplete reduction of oxoammonium cations) is not observed.

In order to demonstrate the encapsulation abilities of P(TEMPO^+^-*r*-OEGMA) hydrogels, we have designed a proof-of-concept experiment by taking opportunity of the presence of positively charged units in the oxidized P(TEMPO^+^-*r*-OEGMA) hydrogels to encapsulate a negatively charged drug, namely ASA, commonly known as aspirin. In a first step, the equilibrium swelling of the P(TEMPO^+^-*r*-OEGMA) hydrogel was realized with aspirin (0.1 g/L) dissolved in 0.1 M aqueous NaClO_4_ solution (at this pH close to 7 the aspirin is mainly negatively charged since its carboxylic group is in the carboxylate anion form). After the swelling step, the hydrogel was rinsed with water and a small amount of the 1 M NaClO_4_ solution was deposited on top of the aspirin loaded P(TEMPO^+^-*r*-OEGMA) hydrogel that was further equilibrated for 12 h (Figure 2a). Afterwards, the supernatant of the hydrogel has been analyzed by mass spectrometry and no aspirin molecules were detected confirming their strong electrostatic encapsulation in the hydrogel and the fact that no aspirin molecules are diffusing out of the hydrogel with time (Figure 2a).

**Figure 3:**
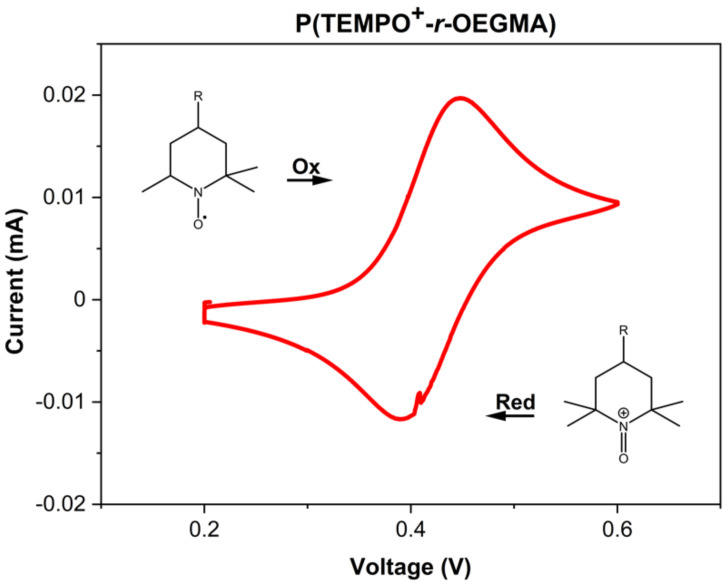
Cyclic voltammetry of P(TEMPO^+^-*r*-OEGMA) hydrogels. Analyses conducted in 0.1 M NaClO_4_ in distilled water at a carbon electrode. Scan rate = 5 mV s^−1^.

In order to electrochemically address the aspirin-loaded P(TEMPO^+^-*r*-OEGMA) hydrogel, a small piece (2 mm^2^) of the hydrogel was cut and brought in direct contact with a printed carbon electrode to perform the electrochemical reduction of TEMPO^+^ into TEMPO (Figure S1). Cyclic voltammetry measurements (CV) for the P(TEMPO^+^-*r*-OEGMA) hydrogel loaded with aspirin show clearly the reversible reduction of the oxoammonium cations into nitroxide radicals (Figure 3).

In order to demonstrate the release of aspirin molecules during the reduction step, the supernatant aqueous solution above the hydrogel was analyzed by mass spectrometry and the presence of aspirin molecules released from the hydrogel was detected (Figure 2b, and green mass spectrum in Figure S2). In a second step, the electrochemical oxidation of the P(TEMPO-*r*-OEGMA) hydrogel into P(TEMPO^+^-*r*-OEGMA) was performed and followed by CV (Figure 3). Once again, the supernatant aqueous solution above the hydrogel was analyzed by mass spectrometry but this time no aspirin molecules were detected (Figure 2b and blue mass spectrum in Figure S2). In order to demonstrate that the scenario depicted in Figure 2b is operating and that no aspirin molecules remain trapped into the hydrogel because of e.g., incomplete reduction of oxoammonium cations (see sketch depicted in Figure 2c), UHPLC has been used to determine the amount of aspirin released in the supernatant of the P(TEMPO^+^-*r*-OEGMA) hydrogels after the electrochemical reduction. The determined concentration of aspirin molecules was 1372 µM (247 ppm), for a total surface of 6.9 mm^2^ (surface of the electrode) and a cut gel surface of 2 mm^2^. The further calculation indicated that 100% of aspirin molecules were released in the whole supernatant, proving the efficiency of the electrochemically triggered release process in agreement with the sketch depicted in Figure 2b.

### 3.3. P(TEMPO-r-OEGMA) Hydrogels as Catalytic Scaffolds for the Oxidation of Alcohols

**Figure 4:**
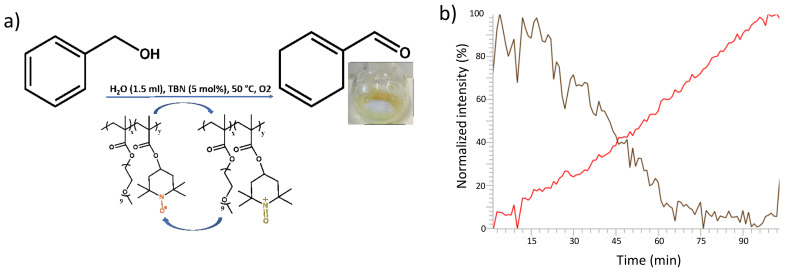
(**a**) Reaction conditions for benzyl alcohol oxidation. (**b**) Evolution of the normalized integration of the infrared spectra of benzylic alcohol and benzaldehyde during the oxidation reaction of benzyl alcohol (black curve) into benzaldehyde (red curve) over time.

The catalytic activity of P(TEMPO-*r*-OEGMA) hydrogels has been determined by monitoring the oxidation of benzylic alcohol under aerobic condition (Figure 4a). Practically, the reaction was started by mixing in a 10 mL round-bottom flask the dry gel (153 mg) and all the reactants (benzylic alcohol, 0.52 mL; tert-butyl nitrite (TBN) as a metal-free co-catalyst, 5 mol% and water, 1.5 mL) together and stirring for 2 h at room temperature. In a second step, the temperature was raised to 50 °C to start the oxidation reaction for 4 h. Then, the reaction was stopped by let it cool down to room temperature for 30 min and the supernatant of the gel was analyzed.

GC-MS analysis of the products of the reaction confirmed quantitative benzylic alcohol conversion into benzaldehyde. It was found that 100% of benzyl alcohol was converted into benzaldehyde (Figure S3). The reaction conditions and corresponding conversion rates of different experiments for the oxidation of benzyl alcohol to benzaldehyde in water based on TEMPO catalysts have been summarized in Table 1.

Compared with other representative experimental conditions, without the addition of transition metal co-catalysts, our P(TEMPO-*r*-OEGMA) hydrogels still maintain competitive catalytic efficiency and conversion rate. Such a high catalytic efficiency of the P(TEMPO-*r*-OEGMA) hydrogel could be mainly due to its composition allowing an important and uniform uptake of benzylic alcohol inside the hydrogel. In this respect, a homogenous system is observed when the P(TEMPO-*r*-OEGMA) hydrogel is added with the reactants and equilibrated. Benzylic alcohol was absorbed inside the matrix and as the reaction starts to proceed, a supernatant appears on top of the hydrogel consisting of benzaldehyde that phase separates from the hydrogel. This last characteristic feature is very interesting since it allows the easy recovery of the product of the reaction and the easy recycling of the TEMPO catalyst.

In order to obtain more information about the kinetics of the oxidation reaction of benzylic alcohol into benzaldehyde, an in situ infrared monitoring of the reaction was realized. Practically, the reaction was performed using the same experimental conditions as described above in the presence of an infrared probe immersed in the reaction medium. The recording of the infrared probe was started when temperature was raised to 50 °C to start the oxidation reaction. The formation of benzaldehyde is followed in the infrared spectra by the peaks at 1700, 1167 and 1204 cm^−1^ corresponding to vibrations associated with the aldehyde function. On the other hand, the peaks corresponding to the benzylic alcohol molecule in the area between 1605 and 1660 cm^−1^ are linearly vanishing with time. The recorded data allow the deduction of two main trends corresponding to a linear disappearance of benzylic alcohol and a concomitant linear formation of benzaldehyde over time (Figure 4). Moreover, they also indicate the complete conversion of benzylic alcohol into benzaldehyde after 90 min (Figure 4). Those results indicate a zero order for the kinetics of the reaction as is often the case for diffusion limited reactions due to the presence of a gel [34,35].

## 4. Conclusions

Redox-responsive hydrogels represent a highly interesting and versatile class of materials for loading and release applications in the biomedical field. In this paper, a new class of redox-responsive hydrogel is developed by combining TEMPO stable nitroxide radicals and OEGMA. TEMPO groups can undergo reversible oxidation-reduction reactions that lead to redox activity while OEGMA groups are hydrophilic and allow water swelling to obtain hydrogels. In this contribution, we have taken advantage of the positive charge present on the oxidized form of TEMPO (oxoammonium cations in TEMPO^+^) to electrostatically complex a negatively charged aspirin molecule. We have demonstrated that aspirin is tightly bound to TEMPO^+^ and cannot diffuse out the hydrogel. However, when TEMPO^+^ groups are electrochemically reduced into TEMPO radicals, the electrostatic interaction between aspirin and TEMPO^+^ disappears which allows the release of the aspirin molecule from the hydrogel. Furthermore, by using UHPLC, we have demonstrated that aspirin can be quantitatively released from the hydrogel. Finally, it should be pointed out that a very simple carbon printed electrode has been used to electrochemically trigger our hydrogels, proving that this set-up could be easily utilized for real-life applications. Finally, we have demonstrated the efficiency of our hydrogels as TEMPO-based catalysts for the oxidation of benzylic alcohols to the corresponding aldehydes under aerobic conditions. Moreover, the final products are easily separated from the hydrogel starting materials without using toxic organic solvents or complicated and costly processes.

## Figures and Tables

**Table 1:** Different reaction conditions for the oxidation of benzyl alcohol into benzaldehyde in water with 2,2,6,6-tetramethyl-1-piperidinyloxy (TEMPO)-based catalysts.

Entry	TEMPO [mol%]	Co-Catalyst	Condition	T (°C)	t (hour)	Conversion (%)	Reference
1	0.2	TBN *	O_2_	50	1.5	100	This work
2	0.2	TBN	O_2_	50	4	100	[31]
3	0.3	NaNO_2_, DBDMH *	Air (0.9 MPa)	80	1.5	99.8	[28]
4	5	Cu, K_2_CO_3_	Air	40	24	100	[32]
5	0.68	Co	Air	RT	0.5	98	[33]

* DBDMH: 1,3-dibromo-5,5-dimethylhydantoin, TBN: tert-butyl nitrite

## Data Availability

The data presented in this study are available on request from the corresponding author.

## Supplementary Materials

The following are available online at https://www.mdpi.com/article/10.3390/polym13081307/s1, Figure S1: Experimental set-up for the electrochemical reduction of P(TEMPO^+^-*r*-OEGMA) hydrogels, swollen in aqueous NaClO_4_ and loaded with aspirin using the printed carbon electrode method (PCE); Figure S2: Mass spectroscopy analysis of the supernatant for the oxidation step (blue curve), reduction step (green curve) and the aspirin reference molecule (orange curve); Figure S3: GC-MS spectrum of the solution obtained after oxidation of benzylic alcohol into benzaldehyde.
