# Supplementary material for: Application of Redox-Responsive Hydrogels Based on 2,2,6,6-Tetramethyl-1-Piperidinyloxy Methacrylate and Oligo(Ethyleneglycol) Methacrylate in Controlled Release and Catalysis
Source: Polymers (Basel). 2021 Apr 16;13(8):1307. doi: 10.3390/polym13081307 (PMC8073720; doi:10.3390/polym13081307)
Supplement: Supplementary file 1 [file polymers-13-01307-s001.zip › polymers-1168803-supplementary.pdf]

# Electronic Supplementary Information Section

# Application of redox-responsive hydrogels based on 2,2,6,6-tetramethyl-1-piperidinyloxy methacrylate and oligo(ethyleneglycol) methacrylate in controlled release and catalysis

**Miriam Khodeir [1], He Jia [1], Alexandru Vlad[1] and Jean-François Gohy [1,*]**

[1] Institute of Condensed Matter and Nanosciences (IMCN), Bio- and Soft Matter (BSMA), Université catholique de Louvain, Place L. Pasteur 1, B-1348 Louvain-la-Neuve, Belgium
* Correspondence: jean-francois.gohy@uclouvain.be

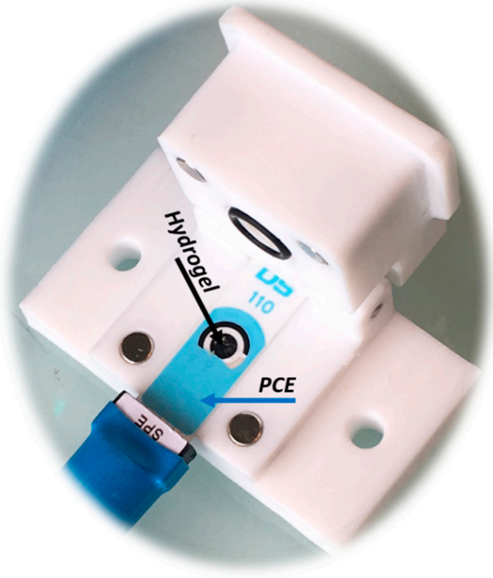


**Figure S1.** Experimental set-up for the electrochemical reduction of P(TEMPO$^+$-*r*-OEGMA) hydrogels, swollen in aqueous $NaClO_4$ and loaded with aspirin using the printed carbon electrode method (PCE).

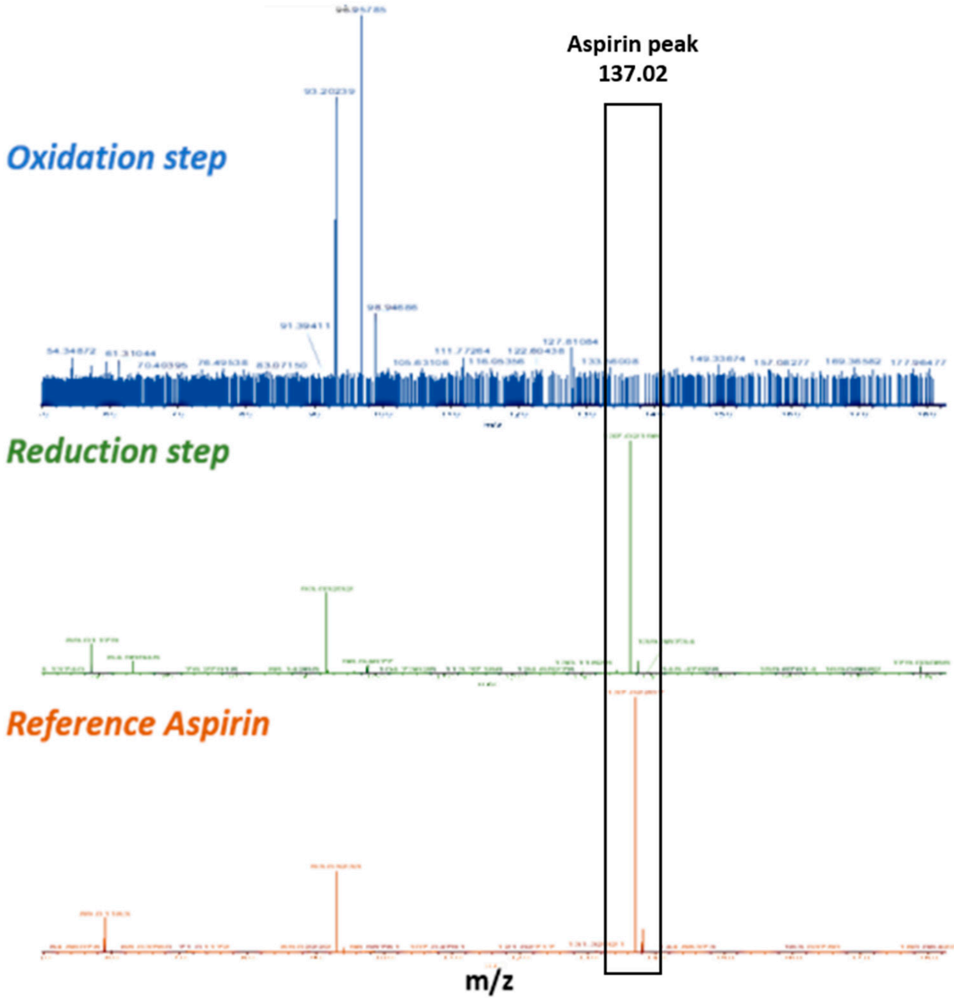


**Figure S2.** Mass spectroscopy analysis of the supernatant for the oxidation step (blue curve), reduction step (green curve) and the aspirin reference molecule (orange curve).

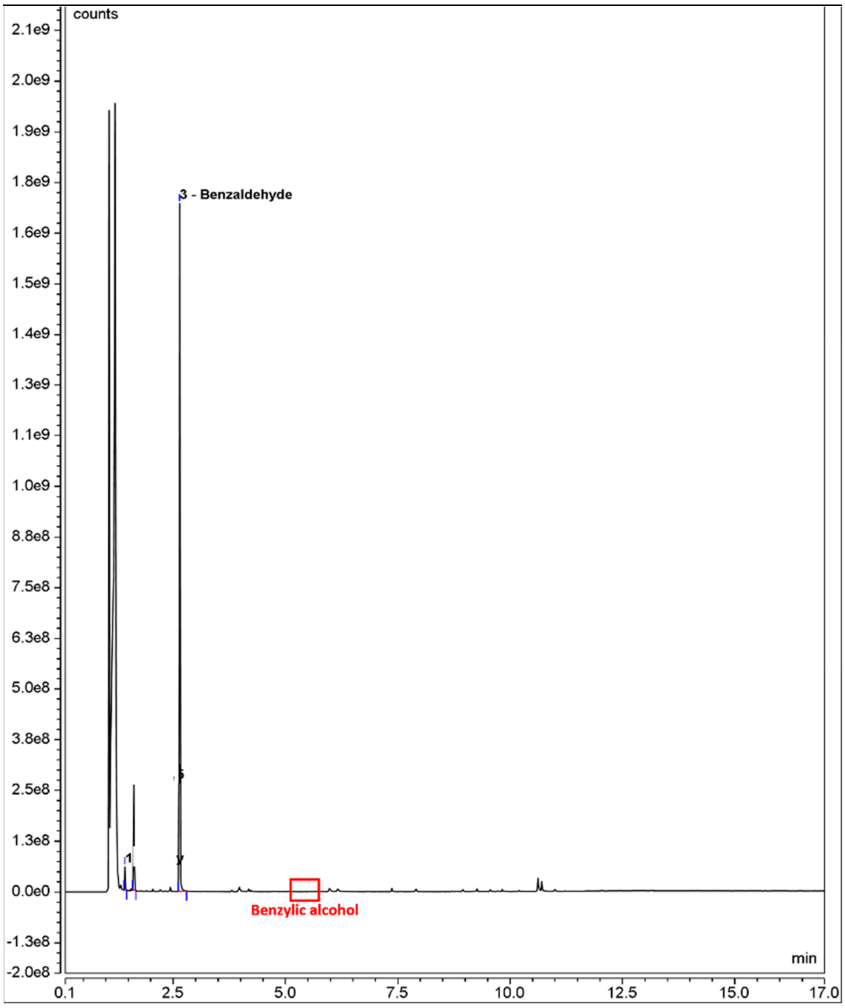


**Figure S3.** GC-MS spectrum of the solution obtained after oxidation of benzylic alcohol into benzaldehyde.
